# Association of *IL7* rs16906115 Polymorphism with Immune-Related Adverse Events in Patients with Advanced Lung Cancer Undergoing Immunotherapy

**DOI:** 10.3390/jcm15041486

**Published:** 2026-02-13

**Authors:** Andrea González-Hernández, Guillermo Paz-López, Beatriz Martínez-Gálvez, Felipe Vaca Paniagua, Isabel Barragán, Elisabeth Pérez-Ruiz, José Carlos Benitez, Antonio Rueda-Dominguez, Javier Oliver

**Affiliations:** 1 Facultad de Medicina, Universidad de Málaga (UMA), 29071 Malaga, Spain; 2 Laboratorio de Investigación Traslacional en Cáncer de Cabeza y Cuello, Centro de Investigación y Terapias Avanzadas del Cáncer (CITAC), 29010 Malaga, Spain; 3 Estructura Común de Apoyo a la Investigación de Bioinformática, Instituto de Investigación Biomédica de Málaga-Plataforma BIONAND (IBIMA-Plataforma Bionand), 29590 Malaga, Spain; 4 Servicio de Oncología Médica, Hospital Virgen de la Victoria, Grupo B-05 Instituto de Investigación Biomédica de Málaga-Plataforma BIONAND (IBIMA-Plataforma Bionand), 29010 Malaga, Spain; 5 Laboratorio Nacional en Salud: Diagnóstico Molecular y Efecto Ambiental en Enfermedades Crónico-Degenerativas, Facultad de Educación Superior Iztacala, Tlalnepantla 54090, Mexico; 6 Unidad de Biomedicina, Facultad de Educación Superior, Universidad Nacional Autónoma de México, Tlalnepantla 54090, Mexico; 7 Servicio de Oncología Médica, Hospital Regional de Málaga, Grupo B-05 Instituto de Investigación Biomédica de Málaga-Plataforma BIONAND (IBIMA-Plataforma Bionand), 29010 Malaga, Spain

**Keywords:** pharmacogenetics, immunotherapy, adverse events, non-small cell lung cancer, personalized medicine, *IL7* polymorphism, biomarkers

## Abstract

**Background**: Immune checkpoint inhibitors (ICIs) have revolutionized the treatment of advanced non-small cell lung cancer (aNSCLC). However, immune-related adverse events (irAEs) remain a clinical challenge in this context. Genetic variants acting as cis-eQTLs may predict toxicity risk, thereby enabling personalized treatment. Specifically, the interleukin *7* (*IL7*) rs16906115 variant has recently been implicated in ICI-related toxicity in other malignancies, like melanoma, although its role in lung cancer remains less defined. We investigated the association between the *IL7* rs16906115 polymorphism, immune-related adverse events (irAEs), and survival outcomes in patients with aNSCLC receiving ICIs. **Methods**: This retrospective cohort study analyzed 153 patients with aNSCLC treated with ICIs (2018–2023) at two centers in Spain. The final analytical cohort included 124 patients with complete clinical follow-up. *IL7* rs16906115 genotyping was performed using TaqMan assays. Associations between genotypes/alleles, irAEs, and survival (PFS/OS) were evaluated using logistic regression and Kaplan–Meier analysis. A clinical–genetic predictive model was developed. **Results**: The A allele frequency was 8.5%. Carriers of the A allele (AG/AA genotypes) had significantly higher irAEs rates compared to GG homozygotes (OR = 3.77, 95% CI: 1.16–12.6, *p* = 0.0081). The association remained significant after multivariable adjustment (OR = 4.64, 95% CI: 1.50–17.2, *p* = 0.0203). Crucially, A-allele carriers exhibited significantly shorter Progression-Free Survival compared to non-carriers (median 6.6 vs. 10 months, *p* = 0.0029). The combined clinical–genetic model achieved moderate predictive performance for toxicity (AUC = 0.67, 95% CI: 0.56–0.78) compared to clinical-only models (AUC = 0.57), stratifying patients into moderate- and high-risk groups, respectively. **Conclusions:** The *IL7* rs16906115 polymorphism is a potential pharmacogenetic biomarker for predicting adverse events in aNSCLC immunotherapy. These findings identify the *IL7* rs16906115 polymorphism as a candidate biomarker, suggesting its potential utility as an exploratory tool for risk stratification that warrants further validation.

## 1. Introduction

Lung cancer remains the leading cause of cancer-related mortality worldwide, with most cases being diagnosed at the metastatic stage. Immune checkpoint inhibitors (ICIs) targeting the Programmed Cell Death Protein 1 (PD-1)/Programmed Death-Ligand 1 (PD-L1) axis have revolutionized the treatment of advanced non-small cell lung cancer (aNSCLC), significantly improving durable responses and survival outcomes [1]. In current clinical practice, ICIs are frequently administered in combination with chemotherapy, which, while enhancing therapeutic efficacy, also increases the complexity of toxicity management [2,3].

Adverse events (AEs) associated with chemo-immunotherapy include both the conventional toxicities of cytotoxic chemotherapy, such as myelosuppression, nausea, fatigue, and neuropathy, and immune-related adverse events (irAEs) induced by ICIs. IrAEs arise from the overactivation of the immune system and can affect virtually any organ system, including the skin, gastrointestinal tract, lungs, and endocrine glands [2]. The incidence of irAEs varies widely, with up to 70% of patients experiencing some form of toxicity and severe irAEs (grades 3–5) occurring in 10–20% of cases [3]. This dual mechanism of toxicity necessitates rigorous clinical vigilance because these complications often require treatment interruption, immunosuppressive therapy, or permanent discontinuation of ICIs, potentially compromising their therapeutic efficacy [4].

Despite advances in understanding the mechanisms underlying irAEs, reliable predictive biomarkers remain elusive [5,6]. Current candidates, such as PD-L1 expression and tumor mutational burden (TMB), are used to guide treatment selection but do not reliably correlate with the risk of developing toxicity [7]. Genetic polymorphisms in immune-related genes have emerged as promising predictors of immune-related adverse events (irAEs). Variants in genes such as *Cytotoxic T-Lymphocyte Antigen 4* (*CTLA-4*), *Programmed Cell Death 1* (*PDCD1*), which encode pivotal immune checkpoint receptors that maintain self-tolerance and *Interleukin-7* (*IL-7*) have been associated with autoimmune diseases, cancer susceptibility, and treatment-related toxicities [4,5,8].

IL-7, a cytokine critical for T cell development and homeostasis, plays a central role in the regulation of immune responses [9]. Previous studies have linked the IL7 pathway to autoimmune diseases, such as multiple sclerosis and type 1 diabetes, in which it modulates T cell activity and cytokine production [10,11]. Notably, IL-7 signaling enhances T-cell survival, proliferation, and effector function, which are key mechanisms underlying ICI efficacy [12]. However, dysregulated IL-7 signaling may also contribute to immune overactivation, potentially increasing the risk of irAEs [13]. The *IL7* gene variant rs16906115, located in an intronic regulatory region, functions as a cis-eQTL in whole blood and lymphoid tissues, where it has been implicated in the modulation of IL-7 expression and immune function [14].

Although this variant has recently been explored as a predictor of ICI-related toxicities, with studies reporting positive associations between melanoma, lung cancer and other solid tumors and irAE development [15,16,17,18,19], its role in aNSCLC requires further evaluation across diverse populations to validate its clinical significance. Genetic effect sizes may differ due to ethnic diversity, disease heterogeneity, and treatment context [20].

In this context, we investigated the association between the *IL7* rs16906115 polymorphism and the development of irAEs in a cohort of Spanish patients with aNSCLC treated with ICIs. We hypothesized that carriers of the A allele (AG/AA genotypes) are predisposed to an increased risk of irAEs owing to enhanced IL-7-mediated immune activation. Furthermore, we explored the integration of this genetic variant into a clinical–genetic predictive model and evaluated its impact on survival outcomes to optimize patient stratification and monitoring in real-world clinical settings.

## 2. Materials and Methods

### 2.1. Study Design and Population

We conducted a retrospective multicenter cohort study involving 153 patients with advanced or metastatic non-small cell lung cancer (aNSCLC) treated with immune checkpoint inhibitors (ICIs) between January 2018 and December 2023. The study was conducted at two tertiary care centers in Spain: Virgen de la Victoria University Hospital and Regional Hospital of Málaga. The inclusion criteria were as follows: (1) histologically confirmed NSCLC, (2) advanced or metastatic disease (Stage IIIB–IV), (3) treatment with anti-PD-1/PD-L1 inhibitors, and (4) availability of high-quality genomic DNA (gDNA) samples. Exclusion criteria were prior immunotherapy, concomitant active autoimmune diseases, and incomplete clinical follow-up data. Although 153 patients were initially genotyped, the final analytical cohort for adverse event (irAEs) association comprised 124 clinically informative patients. Twenty-nine patients were excluded from the primary outcome analysis due to missing or non-validated toxicity data.

### 2.2. Clinical Data and Adverse Event Assessment

Clinical and demographic data were retrospectively extracted from electronic medical records. The variables included age, sex, smoking status, histological subtype, PD-L1 expression (categorized as <1%, 1–49%, or ≥50%), ECOG performance status, treatment regimens (ICI monotherapy vs. chemo-immunotherapy), and treatment response. Adverse events were systematically evaluated and graded according to the Common Terminology Criteria for Adverse Events (CTCAE) version 5.0. Both immune-related AEs (irAEs) and conventional toxicities were recorded.

Immune-related adverse events (irAEs) were defined as adverse events with a potential immunological basis that required close monitoring, corticosteroid, or other immunosuppressive treatments. The diagnosis was confirmed through a combination of clinical assessment, laboratory testing, and imaging studies (with biopsy confirmation when clinically indicated), ensuring that the events were not attributable to tumor progression, concurrent infection, or other concomitant medications. The assessment period for irAEs spanned from the first ICI dose to 90 days after the last dose was administered. In cases of chemo-immunotherapy, toxicities clearly attributable to chemotherapy (e.g., immediate nausea, neutropenia without autoimmune features) were excluded from the primary endpoint analysis based on clinical presentation and timing. To ensure consistency and reduce bias, all AEs were reviewed by two independent investigators blinded to the genotyping results. Toxicity was stratified into no toxicity (grade 0) low-grade (grades 1–2) and high-grade (grades 3–5) for the severity analysis.

### 2.3. DNA Extraction and IL7 rs16906115 Genotyping

Genomic DNA was isolated from peripheral blood samples using the QIAamp DNA Blood Mini Kit (Qiagen, Hilden, Germany), according to the manufacturer’s protocol. *IL7* rs16906115 genotyping was performed using the TaqMan SNP Genotyping Assay (Assay ID: C__32822267_10 Applied Biosystems, Foster City, CA, USA) on a QuantStudio 12 Flex Real-Time PCR System (Thermo Fisher Scientific, Waltham, MA, USA). Automated allele calling was performed using the QuantStudio Analysis Software v1.5.2. To ensure analytical validity, 10% of the samples were genotyped in duplicate, to achieve 100% agreement.

### 2.4. Statistical Analysis

Hardy–Weinberg Equilibrium (HWE) was assessed using a chi-square test to ensure that the population was in genetic equilibrium. Descriptive statistics were used to characterize the study population, and associations between genotypes/alleles and irAEs were evaluated using the chi-square or Fisher’s exact tests. Odds Ratios (OR) and 95% Confidence Intervals (CI) were calculated using logistic regression analysis. A dominant genetic model (GG vs. AG/AA). This was selected a priori due to the low frequency of the homozygous risk genotype (AA, n = 2), which precluded robust additive or recessive modeling. A multivariable logistic regression model was used to identify potential confounding factors. This model was adjusted for age, sex, and treatment modality (ICI monotherapy vs. chemo-immunotherapy) to ensure the independent predictive value of the genetic variant. To adjust for potential confounders, multivariable Cox proportional hazards regression models were constructed for PFS and OS. Covariates included in the model were age, sex, histology, and ECOG performance status. Missing data were handled using complete-case analysis. Hazard Ratios (HR) and 95% Confidence Intervals (CI) are reported. A predictive risk model was developed by integrating the *IL7* rs16906115 A allele count with clinical variables, including sex, histology, PD-L1 expression, and ECOG status. Potential confounders such as ECOG status and PD-L1 expression were evaluated in univariate analyses but were excluded from the final multivariable model due to a lack of statistical significance, preserving model parsimony. The predictive performance was evaluated using the Area Under the Curve (AUC) from the Receiver Operating Characteristic (ROC) analysis. All statistical analyses were performed using R v4.2.1.

### 2.5. Survival Analysis

Exploratory survival analyses were conducted to evaluate the prognostic impact of *IL7* rs16906115 polymorphism. Overall Survival (OS) was defined as the time from the start of immunotherapy to death from any cause. For OS, patients who were alive at the time of analysis were censored at the date of last contact. For PFS, patients without documented progression or death were censored at the date of the last adequate tumor assessment

Survival curves were estimated using the Kaplan–Meier method, and differences between groups were assessed using the log-rank test. All statistical tests were two-sided, and *p*-value < 0.05 were considered statistically significant.

### 2.6. Ethical Considerations

This study was conducted in accordance with the Declaration of Helsinki and was approved by the Málaga Ethical Committee (reference number 4 February 2022). All participants provided written informed consent before their inclusion in the study.

### 2.7. Use of Artificial Intelligence Tools

During the preparation of this work, the authors used Paperpal and Google Gemini to improve language readability, grammatical correctness, and sentence structure. The tool was used to refine the clarity of the Introduction and Discussion sections. After using this tool, the authors re-viewed and edited the content as needed and took full responsibility for the content of the publication.

## 3. Results

### 3.1. Patient Characteristics

**Figure 1:**
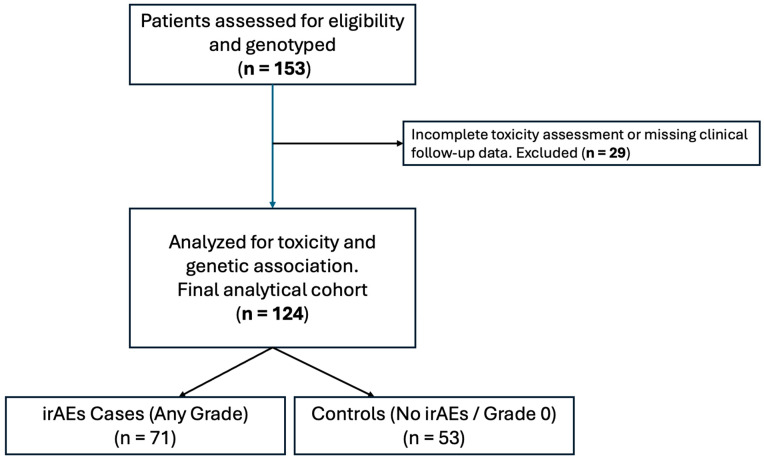
Study flow diagram illustrating patient selection and cohort definitions. From an initially screened population of 153 genotyped patients, 29 were excluded due to incomplete clinical records or undefined toxicity status. The final analytical cohort comprised 124 patients. Patients who did not experience any immune-related adverse events (grade 0, n = 54) served as the control group for the association analyses.

A total of 153 patients with aNSCLC treated with ICIs were included in the initial study population. Patients with no recorded adverse events (grade 0) were classified into the control group. A study flow diagram is provided in Figure 1, detailing the exclusion of 29 patients due to incomplete toxicity data. To assess potential selection bias, we compared the baseline clinical features of the analytical cohort (n = 124) versus the excluded patients (n = 29). No statistically significant differences were observed in age, sex, or histological subtype, confirming that the analytical cohort remains representative of the overall population (Supplementary Table S1). Table 1 provides a detailed summary of the clinical characteristics of the analytical cohort of 124 patients with complete follow-up, stratified by irAEs status (71 with irAEs and 53 without irAEs).

The median age of the cohort was 64 years old. Males comprised 66.7% of the total population, with a balanced distribution between the irAEs (74.6%) and non-irAEs groups (75.5% each). Adenocarcinoma was the most common histological subtype (54.9% of cases). PD-L1 positivity (≥1%) was observed in 83.7% of the cohort, with similar rates between toxicity strata. Pembrolizumab was the most commonly administered ICI (65.4%), followed by Atezolizumab (13.7%) and Nivolumab (6.5%).

### 3.2. IL7 rs16906115 Genotype Distribution and Association with Adverse Events

Among patients, 71 (57.3%) experienced at least one irAE. The toxicity profile was predominantly mild, with low-grade events (grade 1–2) constituting the majority of cases. The most frequently affected organ systems were the skin (21.8%), general symptoms (asthenia, 21.0%), and liver (19.4%). Severe toxicities (Grade 3–4) were uncommon, affecting only 10 patients (8.1%). The detailed frequencies and severity of irAEs by organ system are summarized in Table 2.

*IL7* rs16906115 polymorphism was genotyped in all 153 patients, following the distribution of GG (n = 129, 84.3%), AG (n = 22, 14.4%), and AA (n = 2, 1.3%). The minor allele frequency (MAF) of the A allele was 8.5%, which is consistent with the estimates for the European population. The genotype distribution was in Hardy–Weinberg Equilibrium (*χ*^2^ = 0.12, *p* = 0.73).

The association between *IL7* rs16906115 and irAEs was assessed in 124 patients with clinical information (Table 3). The AG genotype was significantly more frequent in the irAEs group than in the non- irAEs group (18.3% vs. 5.7%; OR = 3.03, 95% CI: 0.88–10.3, *p* = 0.037). Conversely, the GG genotype conferred a protective effect (OR 0.28 [95% CI 0.08–0.94]; *p* = 0.015). At the allele level, the A allele was significantly associated with a 3.71-fold increased risk of irAEs (12.0% vs. 2.8%, *p* = 0.0081). This association remained consistent across treatment modalities and was observed in both ICI monotherapy (OR = 3.58, *p* = 0.045) and chemoimmunotherapy (OR = 3.59, *p* = 0.044) subgroups.

### 3.3. Genetic Models and Multivariable Analysis

Given the rarity of the homozygous variant (n = 2), dominant coding was adopted to preserve the statistical power. Although this approach identified a significant risk group, additive effects could not be excluded and formal model discrimination was not feasible. Multivariable logistic regression analysis adjusted for age and sex confirmed that patients carrying the A allele (AG/AA) had a significantly higher risk of developing irAEs (OR = 4.64, 95%CI: 1.5–17.24, *p* = 0.0203) (Table 3).

### 3.4. Association with Clinical Subgroups and Outcomes

No significant associations were observed between *IL7* rs16906115 genotypes and baseline clinical features such as PD-L1 expression, histological subtype, or sex. However, significant differences were observed in survival outcomes, as detailed below (Table 4).

### 3.5. Predictive Scoring Model for irAEs Risk

**Figure 2:**
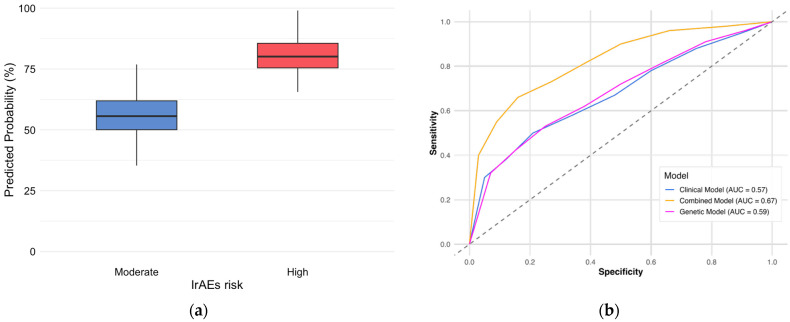
Performance of Predictive Models. (**a**) Risk group stratification probabilities. (**b**) ROC Curves comparing the clinical (blue), genetic (purple), and combined (orange) models for irAEs prediction. The combined model achieved the highest AUC (0.67). The analysis was restricted to immune-related adverse events (irAEs), excluding toxicities attributable to chemotherapy.

A predictive model integrating the *IL7* genotype and key clinical features was developed to stratify the risk of any grade of irAEs. The high-risk threshold (>70%) was identified using the Youden Index to maximize specificity for severe events, while the 30–70% range captured the majority of the cohort. Most patients were stratified into the moderate-risk group (n = 106, 85.5%), with a predicted probability of irAEs between 30% and 70%. Eighteen patients (14.5%) were identified as high-risk (>70% probability). The combined clinical–genetic model achieved an Area Under the Curve (AUC) of 0.67 (95% CI: 0.56–0.78). (Table 5). Numerically, the predictive capacity was improved compared with the clinical-only model (AUC = 0.67 vs. 0.57), although this difference did not reach statistical significance (Figure 2). Given the lack of external validation, this model is presented as an exploratory proof-of-concept for the integration of pharmacogenetic data into clinical stratification.

### 3.6. Association of IL7 Genotype with Survival Outcomes

We further investigated whether the *IL7* variant, in addition to predicting toxicity, was correlated with clinical efficacy. In this exploratory, unadjusted survival analysis, patients were stratified into risk allele carriers (genotypes AG and AA, n = 19) and protective homozygotes (genotype GG, n = 96).

**Figure 3:**
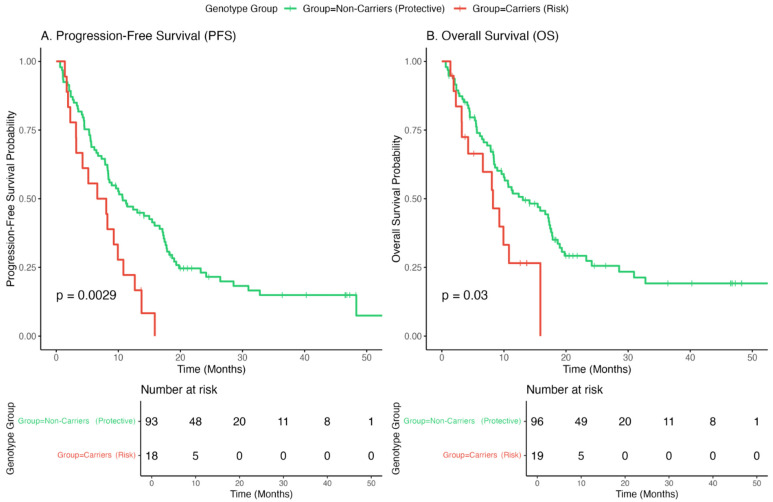
Kaplan–Meier survival analysis stratified by *IL7* rs16906115 status. (**A**) Overall Survival (OS) and (**B**) Progression-Free Survival (PFS) in patients with advanced NSCLC treated with immunotherapy. The analysis compares Risk Allele Carriers (genotypes AG and AA, red line) versus the Protective Group (genotype GG, green line). Carriers of the A allele showed significantly shorter progression-free survival (PFS) (*p* = 0.0029) and overall survival (*p* = 0.03) than non-carriers.

As shown in Figure 3, carriers of the A allele exhibited significantly poorer outcomes than those of the GG group. The median Progression-Free Survival (PFS) was significantly shorter in the risk group (6.6 months; 95% CI: 4.4–8.8) than in the protective group (10.0 months; 95% CI: 8.2–11.9) (log-rank *p* = 0.0029).

Regarding Overall Survival (OS), the analysis also revealed a statistically significant difference (log-rank *p* = 0.03). The risk group showed a median OS of 8.3 months (95% CI: 4.2–NR), whereas the protective group had a median OS of 13.0 months (95% CI: 9.6–17.6 months). These findings indicate that the *IL7* rs16906115 A allele serves not only as a predictor of toxicity but also as a marker of poor prognosis, associated with both earlier progression and reduced overall survival.

To confirm these findings, a multivariable Cox proportional hazards model was constructed, adjusting for age, sex, histology, and ECOG performance status (Supplementary Table S2). The analysis confirmed that the presence of the risk allele was an independent predictor of poor outcomes. Specifically, the risk variant was significantly associated with shorter OS (Adjusted HR = 1.75; 95% CI: 1.09–2.80; *p* = 0.0197) and shorter PFS (Adjusted HR = 1.35; 95% CI: 1.05–1.73; *p* = 0.019), suggesting that the survival disadvantage in these patients is not driven solely by baseline clinical characteristics

## 4. Discussion

### 4.1. Biological Plausibility and Mechanistic Insight

The biological link between the *IL7* rs16906115 polymorphism and ICI induced toxicity is rooted in the fundamental role of this cytokine in T-cell homeostasis. This variant, located in an intronic regulatory region, functions as a cis-eQTL that modulates *IL-7* expression [15,16]. Carriers of the A allele exhibit increased *IL7* transcription, which is essential for the survival, proliferation, and effector functions of CD8+ T cells. Although this immune amplification is key to ICI efficacy, it appears to lower the threshold of immune overactivation. Our findings suggest that the A allele acts as a genetic “trigger” for irAEs independent of the treatment modality (monotherapy vs. chemo-immunotherapy). Notably, the significant association observed in the ICI monotherapy subgroup (OR = 3.58) reinforces that this effect is driven by immune-mediated mechanisms rather than by non-specific chemotherapy toxicity.

Typically, the occurrence of irAEs is often correlated with an improved tumor response in patients treated with ICIs, which is interpreted as a sign of robust immune activation. However, our study revealed a dual detrimental effect of the *IL7* rs16906115 A allele. Carriers of this variant not only experienced higher rates of toxicity but also demonstrated significantly shorter Progression-Free Survival (6.6 vs. 10.0 months, *p* = 0.0029). This observation challenges the widely held clinical paradigm that irAEs serve as a surrogate for therapeutic efficacy. Instead, in this genetic context, toxicity appears uncoupled from the antitumor response, suggesting a distinct pathogenic mechanism driven by non-specific inflammation, which. We hypothesized that the constitutive overexpression of IL-7 in A-allele carriers may lead to a dysregulated pro-inflammatory immune milieu rather than an effective anti-tumor response. Excessive IL-7 signaling could promote the exhaustion of CD8+ T-cells or the activation of lower-affinity T-cell clones, which cause tissue damage (toxicity) without effectively clearing the tumor. Thus, the rs16906115 variant has emerged as a biomarker of unfavorable prognosis, identifying a subgroup of patients at risk of both severe adverse events and early disease progression.

### 4.2. Comparison with Prior Studies and Population Specificity

The association between the *IL7* rs16906115 A allele and irAEs was first established in melanoma patients by Taylor et al. (2022) and Groha et al. (2022) [15,16]. Our results corroborate these findings in aNSCLC, showing a significant independent association with an adjusted Odds Ratio of 4.64 (95% CI: 1.50–17.2, *p* = 0.0203). The minor allele frequency (MAF) in our Spanish cohort was 8.5%, which is slightly lower than the frequencies reported in Northern European populations (~12–15%) but consistent with Mediterranean estimates from the 1000 Genomes Project [21]. This underscores the importance of population-specific validation for clinical implementation, as the effect sizes and genetic backgrounds may vary across ancestries.

### 4.3. Clinical Utility and Predictive Performance

A critical finding of our study was the development of an integrated clinical–genetic risk model. Although the clinical model alone achieved an AUC of 0.57, the addition of the *IL7* genotype increased the predictive performance to an AUC of 0.67 (95% CI: 0.56–0.78). While this numerical improvement did not reach statistical significance, it contributed to a clearer separation of risk groups. In our cohort, this model stratified 16.3% of patients into a “high-risk” group (>70% probability). However, given the moderate discriminative ability and lack of external validation, this classification should be interpreted as a proof-of-concept, underscoring the potential of integrating genetic markers into future multi-dimensional risk stratification models.

Regarding severity, to ensure data robustness, adverse events were systematically evaluated and graded according to the CTCAE v5.0 and reviewed by two independent investigators blinded to the genotyping results. Based on this, no significant association was observed between *IL7* genotype and toxicity grade (Table 3). However, severity analyses pooled immune- and chemotherapy-related toxicities, suggesting that irAE-specific severity effects require stratified evaluations in future studies. Finally, the association was analyzed using a dominant genetic model. Given the low frequency of homozygous risk carriers (n = 2), this coding was primarily chosen to maximize the statistical power. We acknowledge that model discrimination between dominant and additive inheritance was limited by this rarity; thus, the inference of a dominant pattern should be considered provisional pending validation in larger cohorts.

### 4.4. Limitations and Future Directions

This study has several limitations that must be acknowledged. First, the retrospective design may have introduced a selection bias. Second, the sample size (analytical cohort N = n = 124) and low frequency of the homozygous risk genotype (n = 2) limited our statistical power to detect recessive effects. Or perform extensive stratified analyses. Third, we did not perform functional assays such as measuring serum IL-7 levels, which would have provided direct evidence for the proposed mechanism. Furthermore, while patients were recruited from two centers, potential center-level differences in AE ascertainment cannot be fully ruled out. Finally, the predictive scoring model presented here is exploratory. As an internal derivation cohort analysis without external validation or bootstrapping, the reported performance metrics (AUC) may be optimistic. Further studies with larger sample sizes are required to calibrate and validate this risk score. Future research should explore the integration of rs16906115 into polygenic risk scores (incorporating variants in *CTLA4* or *PDCD1*) and multi-omics models. Large-scale prospective studies are required to validate survival findings and determine whether genotype-informed management can reduce the incidence of severe toxicity without compromising antitumor efficacy. Finally, regarding the predictive model, while the integration of the *IL7* genotype improved the AUC compared with clinical features alone, this difference was not statistically significant. Due to sample size constraints and the retrospective design, we could not perform formal external validation or robust calibration analyses. Therefore, the risk stratification proposed in Figure 2b should be interpreted as exploratory risk stratification. Future multicenter studies are required to validate the model’s discrimination ability in independent cohorts before clinical implementation.

## 5. Conclusions

In conclusion, our study identifies the *IL7* rs16906115 polymorphism as a potential pharmacogenetic biomarker associated with irAEs and poorer survival outcomes in Spanish patients with advanced NSCLC treated with immunotherapy. However, given the retrospective design and sample size, these findings should be considered hypothesis-generating. While they offer a promising proof-of-concept for personalized risk stratification, validation in larger, prospective multicenter cohorts is essential to confirm clinical utility before implementation in routine practice.

## Figures and Tables

**Table 1:** Clinical Characteristics of the Study Cohort. Baseline demographic and clinical features stratified by irAE status.

Characteristic	Total Cohort (n = 124)	irAEs YES (n = 71)	irAEs NO (n = 53)	*p*-Value
**Median Age, range (years)**	65 (42–84)	66 (42–83)	64 (50–84)	0.42
**Sex, n (%)**				
Male	93 (75%)	53 (74.6%)	40 (75.5%)	0.89
Female	31 (25%)	18 (25.4%)	13 (24.5%)	
**Histology** **, n (%)**				
Adenocarcinoma	79 (63.7%)	43 (60.6%)	36 (67.9%)	0.58
Squamous	35 (28.2%)	21 (29.6%)	14 (26.4%)	
Other	10 (8.1%)	7 (9.8%)	3 (5.7%)	
**PD-L1 Expression** **, n (%) ^a^**				
<1% (Negative)	25 (20.5%)	16 (22.5%)	9 (17.0%)	0.61
≥1% (Positive)	97 (79.5%)	55 (77.5%)	42 (79.2%)	
**Metastatic Sites** **, n (%) ^b^**				
Brain	15 (12.1%)	9 (12.9%)	6 (11.1%)	0.76
Lung	62 (50%)	34 (48.6%)	28 (51.9%)	0.72
Liver	16 (12.9%)	8 (11.4%)	8 (14.8%)	0.59
Bone	40 (32.2%)	23 (32.9%)	17 (31.5%)	0.87
**Treatment Modality** **, n (%)**				
ICI Monotherapy	41 (33.1%)	25 (35.2%)	16 (30.2%)	0.67
Chemo-Immunotherapy	83 (66.9%)	46 (64.8%)	37 (69.8%)	
**Treatment Line ^a^**				
First Line	78 (62.9%)	47 (60.3%)	31 (39.7%)	0.46
Second Line	39 (31.5%)	20 (51.3%)	19 (48.7%)	
Unknown/Missing	7 (5.6%)	4	3	
**Outcomes** **, n (%)**				
Treatment interruption	93 (75.0%)	59 (84.2%)	34 (61.8%)	**0.004**
Response at 1 year	38 (30.6%)	20 (29.9%)	18 (38.3%)	0.34

Note: A total of 29 patients were excluded from the irAEs event association analysis due to incomplete clinical records or the lack of definitive toxicity grading. ^a^ data for treatment line were unavailable for 7 patients, and PD-L1 expression status was missing for 2 patients. ^b^ Categories are not mutually exclusive. The sum of counts exceeds the total sample size because some patients presented with metastases in multiple sites simultaneously.

**Table 2:** Frequency and severity of immune-related adverse events (irAEs) by organ system in the analytical cohort.

Organ System	Adverse Event	All Grades, n (%)	Grade 1–2 n	Grade 3–4 n
General	Asthenia	26 (21.0%)	24	2
Skin	Rash/Dermatitis	27 (21.8%)	27	0
Hepatic	Hepatitis	24 (19.4%)	23	1
Endocrine	Thyroiditis/Hypothyroidism	10 (8.1%)	10	0
Gastrointestinal	Colitis/Diarrhea	9 (7.3%)	8	1
Musculoskeletal	Arthritis/Arthralgia	10 (8.1%)	10	0
	Myositis	7 (5.6%)	7	0
Renal	Nephritis	8 (6.5%)	7	1
Hematological	Cytopenias	4 (3.2%)	4	0
Respiratory	Pneumonitis	4 (3.2%)	4	0

**Table 3:** Association of *IL7* rs16906115 Genotypes and Alleles with irAEs (n = 124).

Genotype/Allele	irAEs YES (n = 71)	irAEs NO (n = 53)	OR (95% CI)	*p*-Value
**Genotype, n (%)**				
GG (Wild-type)	56 (78.9%)	50 (94.3%)	Reference	-
AG (Heterozygous)	13 (18.3%)	3 (5.7%)	3.03 (0.88–10.3)	0.037
AA (Homozygous Risk)	2 (2.8%)	0 (0.0%)	2.31 (0.10–49.2)	0.21
**Allele Frequency, n (%)**				
G Allele	125 (88.0%)	103 (97.2%)	Reference	-
A Allele (Risk)	17 (12.0%)	3 (2.8%)	3.71 (1.14–12.0)	0.0081
**Dominant Model, n (%)**				
Non-Carriers (GG)	56 (78.9%)	50 (94.3%)	Reference	-
Carriers (AG + AA)	15 (21.1%)	3 (5.7%)	4.64 (1.50–17.2) ^a^	0.0203

^a^ Adjusted OR from multivariable logistic regression, including age, sex, and treatment modality. Severity was graded according to CTCAE v5.0. Low Grade was defined as Grades 1–2, and High Grade was defined as Grades 3–5.

**Table 4:** Association of *IL7* Genotype with Clinical Outcomes. Impact of risk alleles on toxicity severity and survival metrics.

Clinical Feature	Non-Carriers (GG)	Risk Carriers (AG/AA)	*p*-Value
**Toxicity Severity, n (%)**	n = 106	n = 18	
Low Grade (1–2)	98 (92.5.%)	16 (88.9%)	0.62
High Grade (3–5)	8 (7.5.%)	2 (11.1%)	
**Survival Outcomes, median (months)**	n = 96	n = 19	
Progression-Free Survival (PFS)	10.0 (8.2–11.9)	6.6 (4.4–8.8)	0.0029
Overall Survival (OS)	13.0 (9.6–17.6)	8.3 (4.2–NR)	0.03

**Table 5:** Stratification of Patients According to the irAEs Prediction Model.

Model Type	AUC	Sensitivity	Specificity	Risk Stratification Criteria
Clinical Only	0.57	45.1%	66.0%	Age, Sex, Histology, PD-L1
Genetic Only (*IL7*)	0.59	21.1%	94.3%	rs16906115 Genotype
Combined Model	0.67	52.3%	88.6%	Clinical + *IL7* Genotype
Model Type	AUC	Sensitivity	Specificity	Risk Stratification Criteria

## Data Availability

The datasets generated and analyzed during the current study are available from the corresponding author upon reasonable request.

## Supplementary Materials

The following supporting information can be downloaded at: https://www.mdpi.com/article/10.3390/jcm15041486/s1, Table S1: Comparison of baseline characteristics between the analytical cohort and excluded patients.; Table S2: Multivariable Cox Proportional Hazards Analysis for Progression-Free Survival (PFS).

## Abbreviations

The following abbreviations are used in this manuscript.

Abbreviation	Full Term
AEs	Adverse Events
aNSCLC	Advanced Non-Small Cell Lung Cancer
AUC	Area Under the Curve
CI	Confidence Interval
CTCAE	Common Terminology Criteria for Adverse Events
ECOG	Eastern Cooperative Oncology Group
eQTL	Expression Quantitative Trait Locus
HR	Hazard Ratio
ICIs	Immune Checkpoint Inhibitors
IL-7	Interleukin-7
irAEs	Immune-related Adverse Events
MAF	Minor Allele Frequency
NSCLC	Non-Small Cell Lung Cancer
OR	Odds Ratio
OS	Overall Survival
PD-1	Programmed Cell Death Protein 1
PD-L1	Programmed Death-Ligand 1
