# Supplementary material for: Association of IL7 rs16906115 Polymorphism with Immune-Related Adverse Events in Patients with Advanced Lung Cancer Undergoing Immunotherapy
Source: J Clin Med. 2026 Feb 13;15(4):1486. doi: 10.3390/jcm15041486 (PMC12941508; doi:10.3390/jcm15041486)
Supplement: Supplementary file 1 [file jcm-15-01486-s001.zip › jcm-4116857-supplementary.pdf]

Table S1. Comparison of baseline characteristics between the analytical cohort and excluded patients.

Characteristic	Analytical Cohort (n = 124)	Excluded Patients (n = 29)	*p*-Value
**Median Age (years)**	65	64	0.82
**Sex, n (%)**			
Male	94 (75.8%)	20 (69.0%)	0.44
Female	30 (24.2%)	9 (31.0%)	
**Histology, n (%)**			
Adenocarcinoma	79 (63.7%)	17 (58.6%)	0.61
Squamous	40 (32.3%)	12 (41.4%)	
Other	5 (4.0%)	0 (0.0%)	

Table S2. Multivariable Cox Proportional Hazards Analysis for Progression-Free Survival (PFS).

Variable	Hazard Ratio (HR)	95% Confidence Interval	*p*-Value
IL7 Genotype (Risk Allele Carrier)	1.35	1.05–1.73	0.019
ECOG Performance Status (1–2 vs. 0)	1.20	0.78–1.85	0.400
Sex (Female vs. Male)	0.85	0.56–1.29	0.440
Histology (Non-Squamous vs. Squamous)	1.10	0.72–1.68	0.660
